# Systematic Prediction of Scaffold Proteins Reveals New Design Principles in Scaffold-Mediated Signal Transduction

**DOI:** 10.1371/journal.pcbi.1004508

**Published:** 2015-09-22

**Authors:** Jianfei Hu, Johnathan Neiswinger, Jin Zhang, Heng Zhu, Jiang Qian

**Affiliations:** 1 Department of Ophthalmology, Johns Hopkins School of Medicine, Baltimore, Maryland, United States of America; 2 Department of Pharmacology and Molecular Sciences, Johns Hopkins School of Medicine, Baltimore, Maryland, United States of America; 3 The Sidney Kimmel Comprehensive Cancer Center, Johns Hopkins School of Medicine, Baltimore, Maryland, United States of America; 4 Solomon H. Snyder Department of Neuroscience, Johns Hopkins School of Medicine, Baltimore, Maryland, United States of America; 5 Center for High-Throughput Biology, Johns Hopkins School of Medicine, Baltimore, Maryland, United States of America; University of Chicago, UNITED STATES

## Abstract

Scaffold proteins play a crucial role in facilitating signal transduction in eukaryotes by bringing together multiple signaling components. In this study, we performed a systematic analysis of scaffold proteins in signal transduction by integrating protein-protein interaction and kinase-substrate relationship networks. We predicted 212 scaffold proteins that are involved in 605 distinct signaling pathways. The computational prediction was validated using a protein microarray-based approach. The predicted scaffold proteins showed several interesting characteristics, as we expected from the functionality of scaffold proteins. We found that the scaffold proteins are likely to interact with each other, which is consistent with previous finding that scaffold proteins tend to form homodimers and heterodimers. Interestingly, a single scaffold protein can be involved in multiple signaling pathways by interacting with other scaffold protein partners. Furthermore, we propose two possible regulatory mechanisms by which the activity of scaffold proteins is coordinated with their associated pathways through phosphorylation process.

## Author Summary

Despite their importance in the signaling transduction, there is no systematic effort in identifying and characterizing the scaffold proteins in humans. In this work, we predicted scaffold proteins by integrating the available protein-protein interactions and kinase-substrate relationships. The predicted scaffold proteins showed characteristics for known scaffold proteins, suggesting the fidelity of our prediction. More importantly, the systematic prediction of scaffold proteins provides biological insights in the scaffold-mediated signal transduction. We found that scaffold proteins are likely to form complexes, suggesting that scaffold proteins could participate in diverse signaling pathways through the combinatorial interactions among scaffold proteins. Furthermore, the regulation of scaffold proteins’ activities has not been extensively studied. Our bioinformatics analysis proposed that scaffold proteins themselves might be regulated through phosphorylation process.

## Introduction

Protein phosphorylation and dephosphorylation is an important means of protein regulation that occur in both prokaryotic and eukaryotic organisms [1–5]. Phosphorylation of a protein may result in a conformational change in its structure, recruitment of binding partners or change of localization, leading to its activation or deactivation [6,7]. In the context of a signaling pathway, a relay of phosphorylation events could allow the transmission of extracellular signals to intracellular targets. One well-known example is the RAS-ERK pathway, in which a small G-protein RAS activates MAP3K RAF, which then phosphorylates and activates MAP2K MEK1 (MAPKK1). MEK1 then phosphorylates and activates MAPK ERK1/2[8]. Biological systems contain a large number of phosphorylation-related signaling pathways. Many of these signaling pathways share common signaling components and are subject to extensive cross-regulation. The emergence of complex signaling networks prompts the question of specificity, and understanding how individual signals are transduced to arrive at specific outputs is of great importance to the biological community. It is believed that the answer may partially lie in the existence of scaffold proteins.

Scaffold proteins act as “molecular glue”, linking multiple components in a phosphorylation-dependent signaling pathway together to facilitate signal transduction, and as such play a crucial role in the regulation of signaling cascades [8–13]. The scaffold proteins exert their effects through simple tethering of signaling proteins, properly orienting target proteins, or allosteric assembly of pathway components. They can enhance signaling specificity by sequestering proteins, preventing unwanted cross-influence between proteins in different signaling pathways. They can also increase the signaling efficiency by increasing the local concentration of each signaling component. Thus, the knowledge of scaffold proteins can help improve our understanding of the regulation of subcellular signal transduction [14].

Traditional biochemistry approach to identifying scaffold proteins requires multiple steps [15,16], including 1) selection of a candidate as a scaffold protein and the corresponding signaling pathway; 2) testing the protein-protein interactions between the scaffold candidate and the protein members of the selected pathway; and 3) assessment of the enhanced signaling readout of the signaling pathway in the presence of the scaffold candidate [12]. To date, there is no report on a systematic effort to comprehensively identify scaffold proteins. In this work, by taking advantage of the existing extensive datasets of protein-protein interactions (PPIs) and kinase-substrate relationships (KSRs), we developed a statistical approach to predict scaffold proteins. We predicted a large number of potential scaffold proteins, which share many similar characteristics with known scaffold proteins. Interestingly, we discovered that these predicted scaffold proteins are likely to form scaffold complexes and contain more phosphorylation sites than other proteins in human proteome, suggesting that the functionality of the scaffold proteins might be regulated by phosphorylation process.

## Results

### Protein mediators are widespread in signaling networks

**Fig 1:**
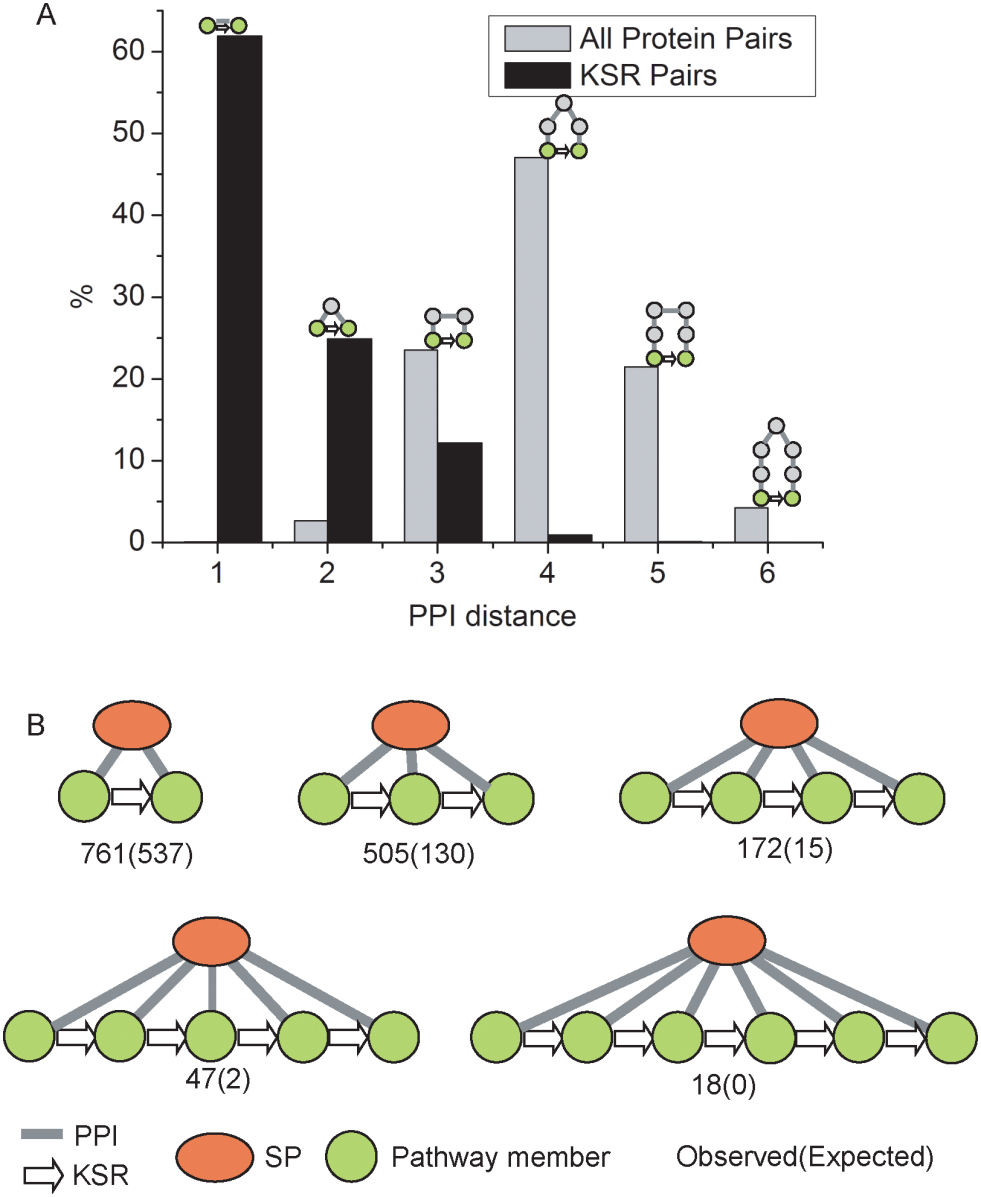
Scaffold proteins are widespread in signaling networks. (*A*) PPI distance of KSR pairs and all human protein pairs. The PPI distance of a protein pair is defined as the shortest distance of the two proteins in PPI network. KSR pairs are significantly enriched in PPI distance = 2. In fact, 24.9% of KSR pairs have PPI distance of 2, while only 2.7% of all human protein pairs have the same PPI distance. (*B*) Network motifs in which one protein interacts with a series of proteins and these proteins form a cascade via KSRs. These network motifs are enriched, suggesting that scaffold proteins are widespread in signaling pathways.

We first construct a composite network, which includes 55,048 protein-protein interactions (PPIs) and 1103 kinase-substrate relationship (KSR) in human [3,5,17]. For a given protein pair, we calculated the shortest distances connecting them in the PPI network (see Methods). A distance of 1 indicates that two proteins directly interact with each other, while a distance of 2 indicates that they do not interact directly with each other, but both interact with a third protein (Fig 1A). Among 1,103 protein pairs with known KSRs, 24.9% of them have a distance of 2 in the PPI network, suggesting that these signaling proteins are likely to interact with a shared protein mediator. In contrast, of the 6.4×10^7^ human protein pairs in the PPI network, only 2.7% have a distance of 2 (Fig 1A). The shortest distance analysis suggested that protein mediators might be widespread among signaling proteins in the phosphorylation networks.

We next examined the network motifs in the composite network, which represent the basic building blocks in a network [18]. The network motif relevant to scaffold proteins is single-input module (SIM), where a single regulator regulates a set of proteins [19]. Here, the single regulator corresponds to a scaffold protein, while the set of proteins are the protein members in a signaling pathway. In our analysis, a SIM is identified if one protein shows PPIs with a set of proteins and the set of proteins form a linear cascade through KSRs (Fig 1B). We observed that the occurrences of the SIMs are significantly enriched as compared to their expected occurrences in the networks, where the PPIs were randomly permutated (Fig 1B). For example, the SIM motif with a cascade length of 5 occurs 47 times; whereas only 2 times is expected in a randomized network (Fig 1B). Both shortest distance and network motif analyses suggest that a scaffold mediator is likely a widely-used mechanism in phosphorylation signaling cascades.

### Prediction of scaffold proteins

**Fig 2:**
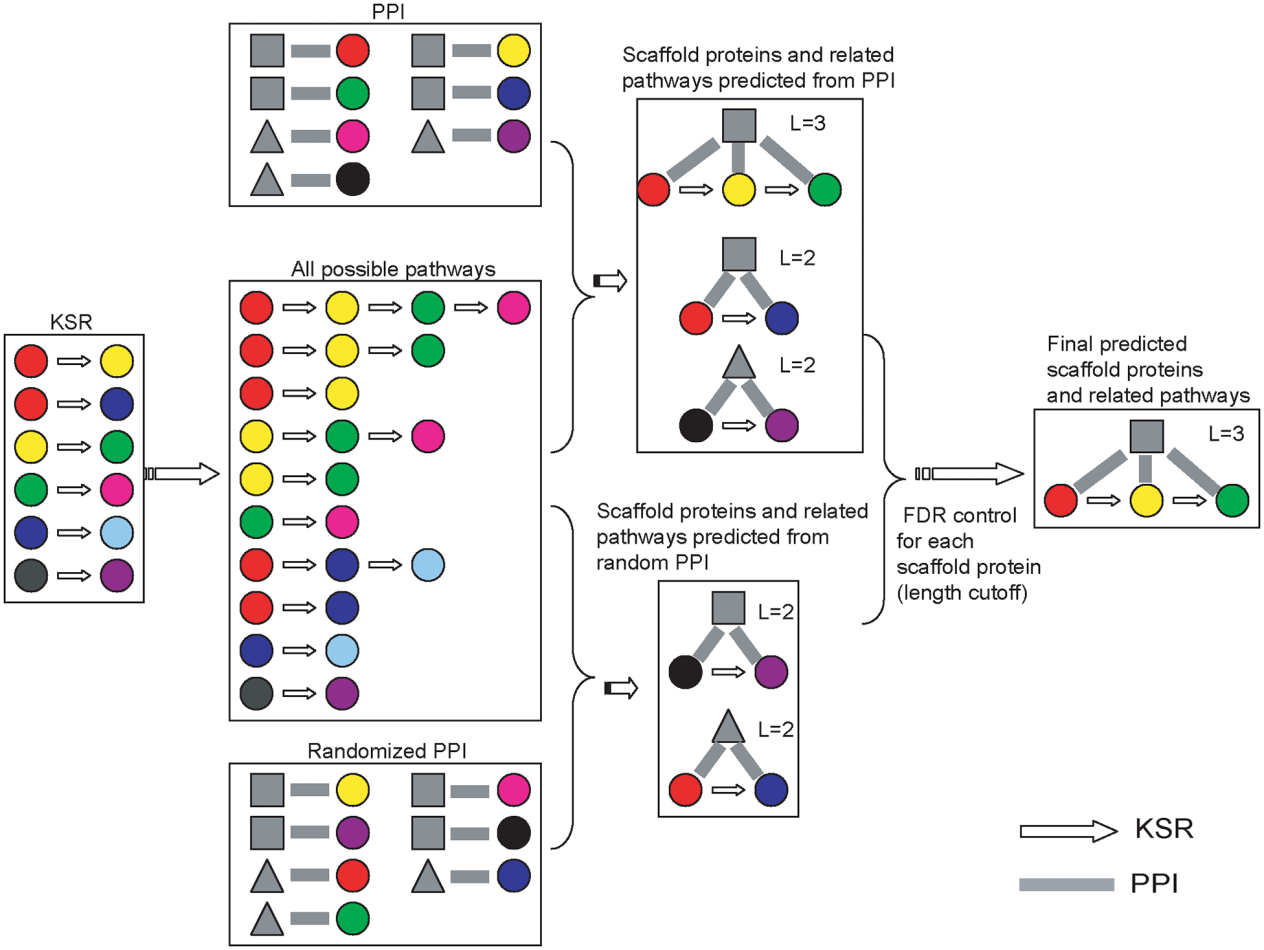
Strategy to predict scaffold proteins. For each potential scaffold protein, we corrected the effect of interaction degree of the protein and the length of associated pathways. We utilized the randomized PPI to assess the significance of a predicted scaffold protein. The random PPI keep the same PPI degree for each protein by randomly selecting two PPI pairs and changing their partners.

In order to predict potential scaffold proteins in phosphorylation signaling cascades, we searched in the composite networks for proteins that show protein-protein interactions with multiple components in KSR networks (Fig 2). Note that in this work we do not distinguish scaffold proteins and adaptors, which are smaller proteins binding only two signaling proteins [20]. The scaffold proteins in this work are simply defined as the protein hubs that interact with multiple members in a signaling pathway.

A stringent requirement was made in predicting potential scaffold proteins by examining whether a given candidate interacts with *all* components in a particular pathway. Here, the pathway is defined as a set of proteins with linear KSRs. For example, if Kinase A phosphorylates Kinase B, and Kinase B phosphorylates Protein C, we constructed a pathway of A → B → C. Some proteins might interact with subset of proteins in the pathway, such as proteins A and B (or proteins B and C) in the pathway. Continuous sub-paths within a long pathway are also considered as separate pathways (such as A → B and B → C). Note that such defined pathways are not necessary to be the same biological pathway as those defined in other databases (e.g., KEGG database)[21].

To assess the statistical significance for predicting scaffold proteins, simulations were performed by permutation of the PPIs, while keeping the interaction degree (i.e., number of interacting partners) for each protein unchanged. For a protein with a PPI degree of *n* and a targeted signaling pathway with length of *l*, we calculated in the permutated networks the chance that a protein with the same PPI degree is predicted as a scaffold protein.

Using 1000 random PPI data to calculate the false discovery rate and choosing 0.01 as the cutoff of false discovery rate, 212 proteins were predicted as scaffold proteins, which are associated with 605 non-redundant phosphorylation pathways. Among the 1,103 known KSRs, 359 of them (33%) are associated with at least one predicted scaffold protein. The resulting network is shown in S1 Fig. The predicted scaffold proteins and their associated pathways are listed in S1 Table.

We then examined whether these scaffold proteins are chosen simply because of their high interaction degrees. Based on the PPI degree distribution, we found that the peak of the distribution locates around 10 (S2 Fig). This distribution is similar to that of known scaffold proteins. This result indicates the prediction of scaffold proteins is unlikely to be an artifact due to their high PPI degrees; whereas we did observed that proteins with high PPI degrees have high possibilities to be scaffold proteins (S3 Fig).

We collected 78 known scaffold proteins for kinase signaling pathways through literature curation (S2 Table). Our prediction recovered 18 of them, yielding a sensitivity of 23%. In contrast, when 212 proteins were selected randomly among the whole human proteome (~24,000 proteins), it is only expected to recover 0.69 known scaffold protein. Therefore, our prediction of scaffold proteins is of > 26-fold enrichment (p<6.9×10^−21^, hypergeometric distribution).

### Validation of scaffold proteins using protein microarrays

To experimentally evaluate the quality of our prediction, we performed kinase reactions on a human proteome array (HuProt), which contains over 17,000 full-length human proteins, in order to comprehensively examine the effects of predicted scaffold proteins [3,22]. The kinase assays were performed by incubating each array with a purified kinase in the presence or absence of its predicted scaffold protein (see methods). Two newly predicted scaffold proteins, activating transcription factor 2 (ATF2) and peptidylprolylcis/trans isomerase, NIMA-interacting 1 (PIN1), were selected for validation. Note that these two scaffold proteins do not contain kinase domain so that they themselves will not directly enhance the phosphorylation activity on the substrates. ATF2 is highly conserved in vertebrates and it is comprised of a C-terminal basic leucine zipper (bZIP) domain and an N-terminal GCN4 central activation domain-like acidic activation domain. This protein can specifically bind the CRE DNA motif to activate downstream transcription. As a peptidyl-prolylcis/trans isomerase, PIN1 catalyzes the cis/trans isomerization of peptidyl-prolyl peptide bonds. It specifically binds to phosphorylated pS/TP motifs to catalytically regulate the post-phosphorylation conformation of its substrates and has a profound impact on key proteins involved in the regulation of cell growth, genotoxic and other stress responses, the immune response, germ cell development, neuronal differentiation, to name a few.

In our dataset, ATF2 was predicted to act as a scaffold protein for kinases of CKII (CSNK2A1) and MAP kinase JNK2 (MAPK9), while PIN1 was predicted to act as a potential scaffold for CKII (S3 Table). We tested whether the predicted scaffold proteins will enhance the phosphorylation signals on the substrates presented on the HuProt array. In order to determine the activity of the purified kinases, a standard dot blot assay was first performed for CKII and JNK2, and both were found to have good activity (S4 Fig). Each HuProt array was incubated with the purified kinase in a standard phosphorylation reaction buffer using ^33^P-γ-ATP as a labeling reagent in the presence or absence of its candidate scaffold protein. To ensure reproducibility, all the kinase reactions were performed in duplicate. Phosphorylation array images were compared side-by-side and each positive hit was identified with the GenePix software and validated by visual inspection. A scaffold protein-dependent substrate was identified with following criteria. First, a true phosphorylated substrate must have a signal intensity greater than 1.5 (see Methods). Second, a positive must be reproducible in the duplicate. Third, a true positive should be found phosphorylated only in the presence of the scaffold protein but not in the absence of the scaffold.

**Fig 3:**
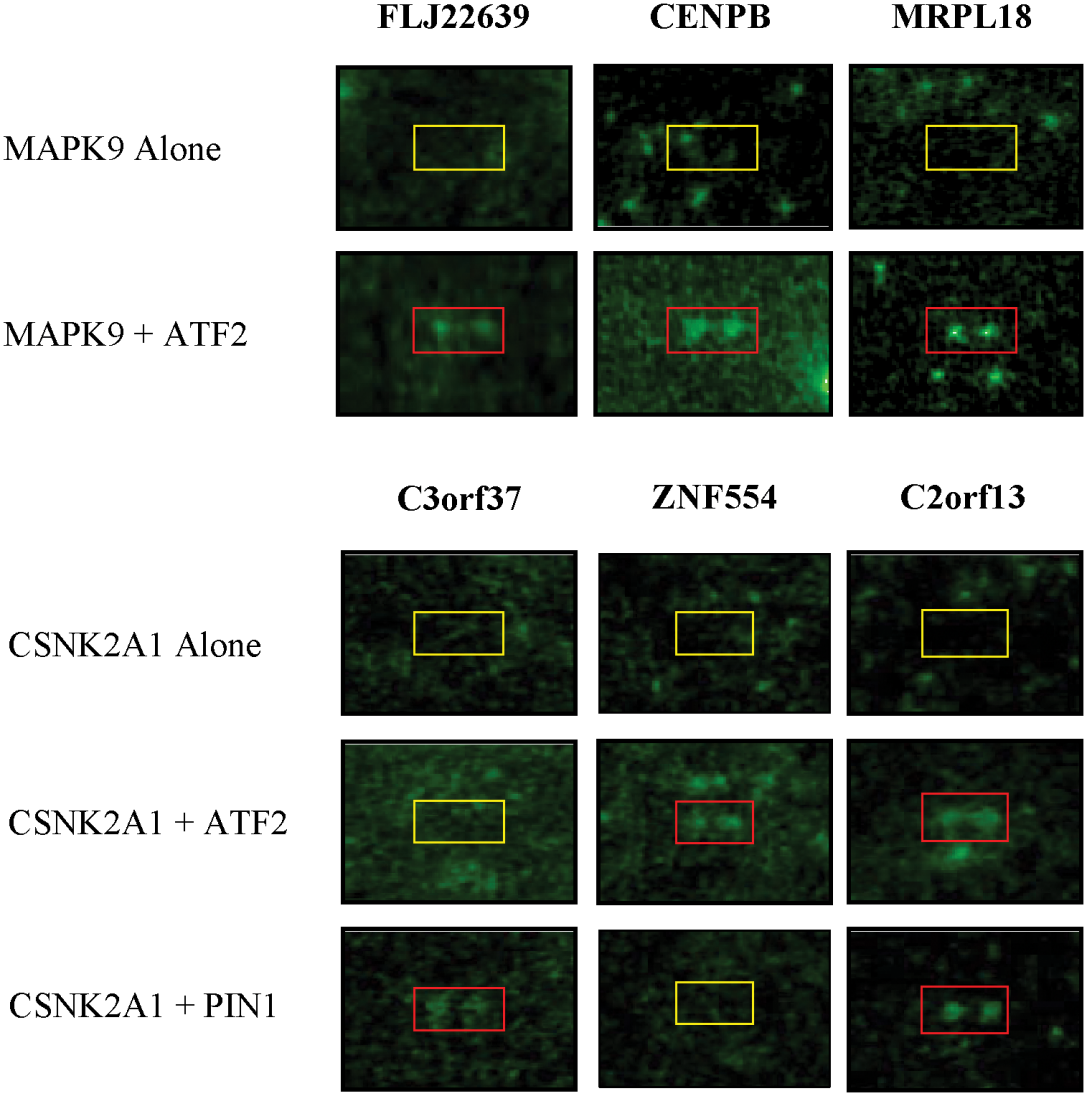
Experimental validations for *CSNK2A1* and *MAPK9*. A human proteome microarray, comprised of 17,000 individually purified human proteins in full-length, was used to perform phosphorylation reactions with CKII (CSNK2A1) and JNK2 (MAPK9) in the presence or absence of their predicted scaffold proteins, ATF2 and PIN1. Phosphorylation signals were detected by exposure of the human proteome microarrays to X-ray film. Positive hits in red boxes were identified by visual inspection.

Using these criteria, 28 scaffold protein-dependent phosphorylation events were discovered between JNK2 and CKII (S4 Table). For example, JNK2 could only phosphorylate FLJ22639, CENPB, and MRPL18 in the presence of its predicted scaffold protein ATF2, suggesting that ATF2 facilitates JNK2 phosphorylation of these substrates (Fig 3). Interestingly, both PIN1 and ATF2 can act as scaffold proteins for the pathway of CKII →C2orf13. However, PIN2 and ATF2 can also act specifically on pathways of CKII → C3orf37 and CKII → ZNF554, respectively. In summary, the successful identification of novel scaffold proteins and scaffold-dependent KSRs strengthens our initial predictions.

### Most scaffold proteins are specific to pathways

**Fig 4:**
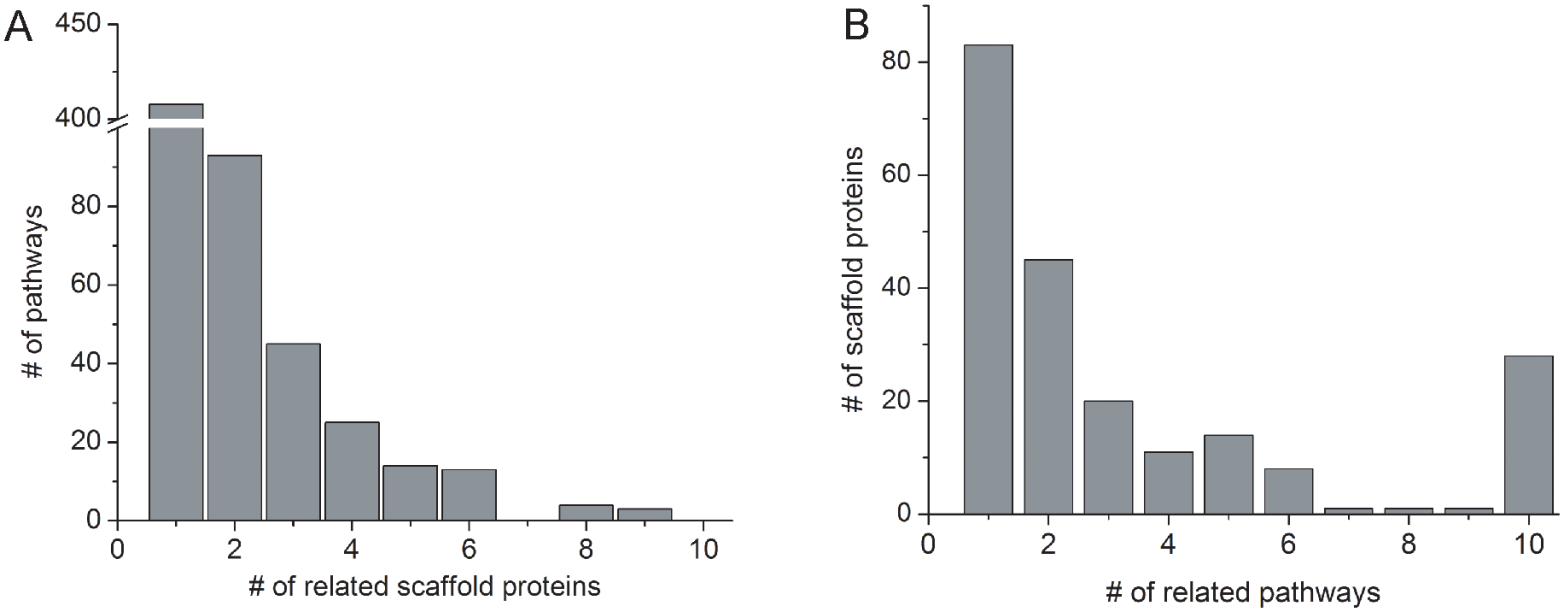
Specificity of scaffold proteins and pathways. (*A*) Number of pathways related to scaffold proteins. 408 pathways (67.4%) are found to be associated with only one scaffold protein. (*B*) Number of scaffold proteins related to pathways. Specifically, 83 scaffold proteins are associated with only one pathways, while 28 scaffold proteins are related to >10 pathways.

Of the 605 scaffold-mediated phosphorylation pathways, 408 (67%) are associated with only one scaffold protein, suggesting that the signaling pathways are likely to be specifically regulated by a single scaffold protein (Fig 4A). On the other hand, 61% of scaffold proteins are associated with more than one pathway, suggesting that these scaffold proteins can participate in multiple pathways (Fig 4B).

Some partially overlapped pathways are involved in different biological processes and can be regulated by different scaffold proteins. For example, one signaling pathway, namely PLK1→WEE1→CDC2→CDC25C, is associated with a scaffold protein PIN1. The pathway is partially overlapped with the pathway of CDC2→CSNK2A1→AKT1, which is associated with scaffold protein Tyrosine-protein phosphatase non-receptor type 1 (PTPN1). Although CDC2 participates in both pathways, the two scaffold proteins might provide specificity to the signaling pathways and prevent possible undesired crosstalk between pathways.

### Characterization of scaffold proteins

**Fig 5:**
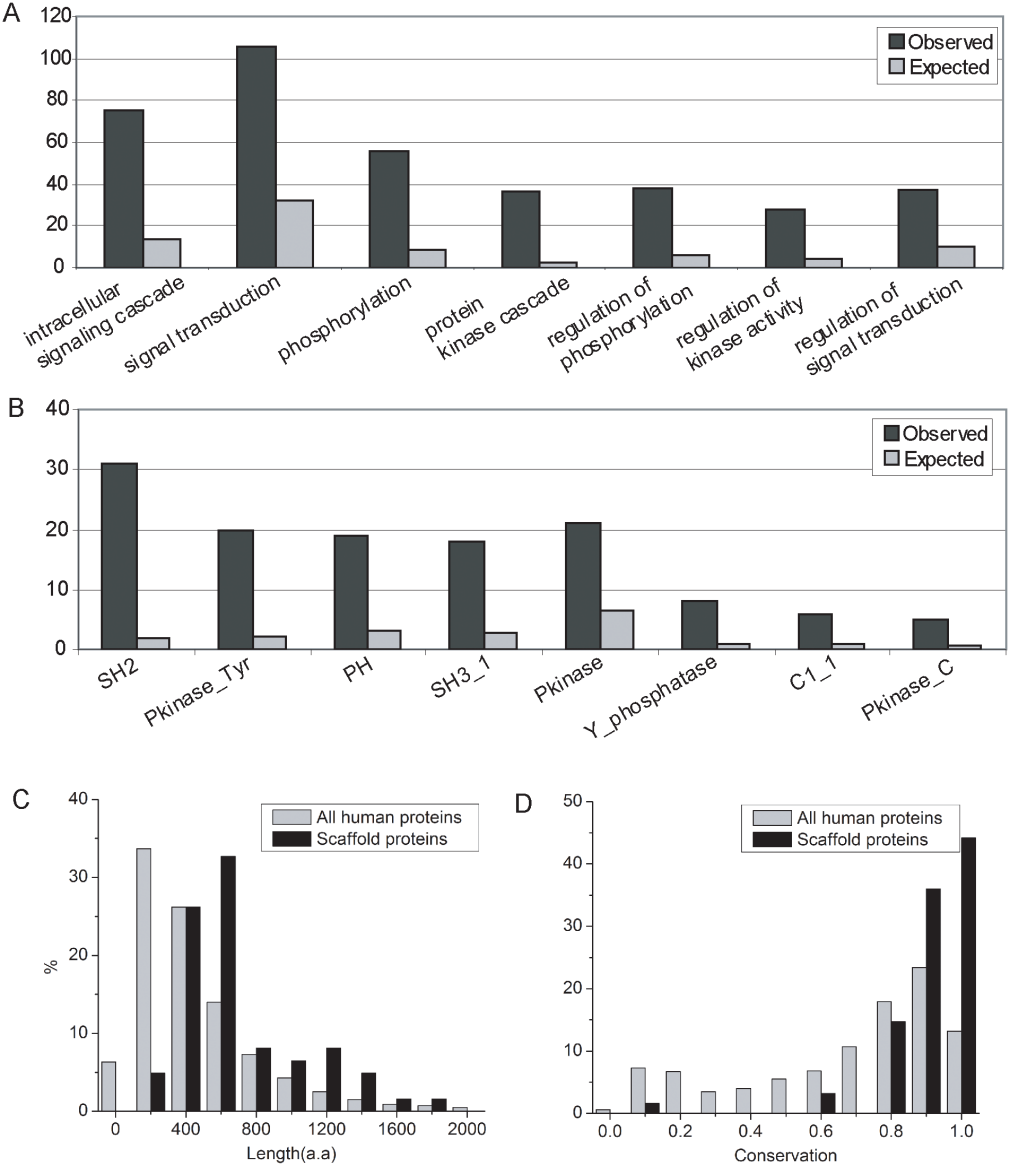
Characterization of scaffold proteins. (*A*) Enriched GO terms for scaffold proteins. (*B*) Enriched protein domains defined by Pfam in scaffold proteins. The GO and Pfam terms are sorted increasingly from left to right by p-value. (*C*) Distribution of protein lengths. (*D*) Distribution of evolutionary conservation.

To further our understanding of the biological process that these scaffold proteins might be involved, we examined the gene ontology (GO) annotation associated with the predicted scaffold proteins. The GO biological process analysis indicates that 106 of the 212 predicted scaffold proteins are associated with the GO term “signal transduction” (p<1×10^−28^, hypergeometric distribution), and that 75 of them are annotated to be related to “intracellular signaling cascade” (p<1×10^−32^, hypergeometric distribution), both over three-fold enrichment than expected (Fig 5A). Furthermore, 38 of predicted scaffold proteins are associated with the GO term “regulation of phosphorylation,” and 36 with “protein kinase cascade.”

To gain molecular insights into how these predicted scaffold proteins might function in signaling cascades, we examined the protein domains encoded by these proteins as defined in Pfam [23]. Compared to the expected occurrence of the corresponding domains, we found several enriched protein domains in these predicted scaffold proteins (Fig 5B). Many of them are known to interact with phosphorylation sites and play a role in signaling cascades, including SH2, SH3, and PH, suggesting that many predicted scaffold proteins are directly involved in kinase signaling. For example, SH2 domains are known to interact with phosphorylated tyrosine sites and regulate the signaling pathways [24,25]. Interestingly, kinase domains are also enriched, such as Pkinase and Pkinase_Tyr, suggesting that some scaffold proteins are kinase themselves. In fact, 18.9% (40/212) of predicted scaffold proteins are kinases, which is consistent to the previous finding that some kinases can act as scaffold proteins [26,27]. However, only 8% (40/518) of kinases in our dataset were predicted as scaffold proteins.

Furthermore, we examined the size of the predicted scaffold proteins. Scaffold proteins are generally large proteins because they need to interact with multiple proteins simultaneously, although some known and predicted scaffold proteins are small proteins as they can form large complexes of polymer, such as ISCU [28]. The comparison between predicted scaffold proteins and the human proteome shows the predicted scaffold proteins are significantly larger than that of background (average 670 residues for scaffold proteins vs. 200 residues for all human proteins) (Fig 5C). This property is partially due to the higher interaction degree of scaffold proteins. If we compared the protein sizes between scaffold proteins and the proteins with similar interaction degrees, their protein sizes showed no significant difference (S5 Fig).

If scaffold proteins are essential for signaling pathways, it is expected that these proteins should be under evolutionary constraint. By comparing the human protein sequences with their mouse counterparts, we calculated the conservation score for each human protein. On average, the predicted scaffold proteins have a very high conservation score of 0.90, while the average conservation score for all human proteins is 0.68 (Fig 5D). In fact, 92% of predicted scaffold proteins have conservation scores larger than 0.8, while we only expect that 47% of human proteins have that level of conservation.

Finally, we examined whether the predicted scaffold proteins were co-expressed with the proteins in their associated pathways. Based on the gene expression data across 18 different biological conditions [29], we calculated the gene expression correlation coefficient between scaffold proteins and all members in the pathways, and found that the correlation coefficients were higher than expected from two randomly selected genes (S6 Fig).

In summary, the above analyses of gene ontology, protein domains, protein sizes, evolutionary conservation, and co-expression clearly set apart the predicted scaffold proteins from the rest of the human proteome by showing the characteristics for the functionality of the scaffold proteins.

### Scaffold proteins tend to form complexes

**Fig 6:**
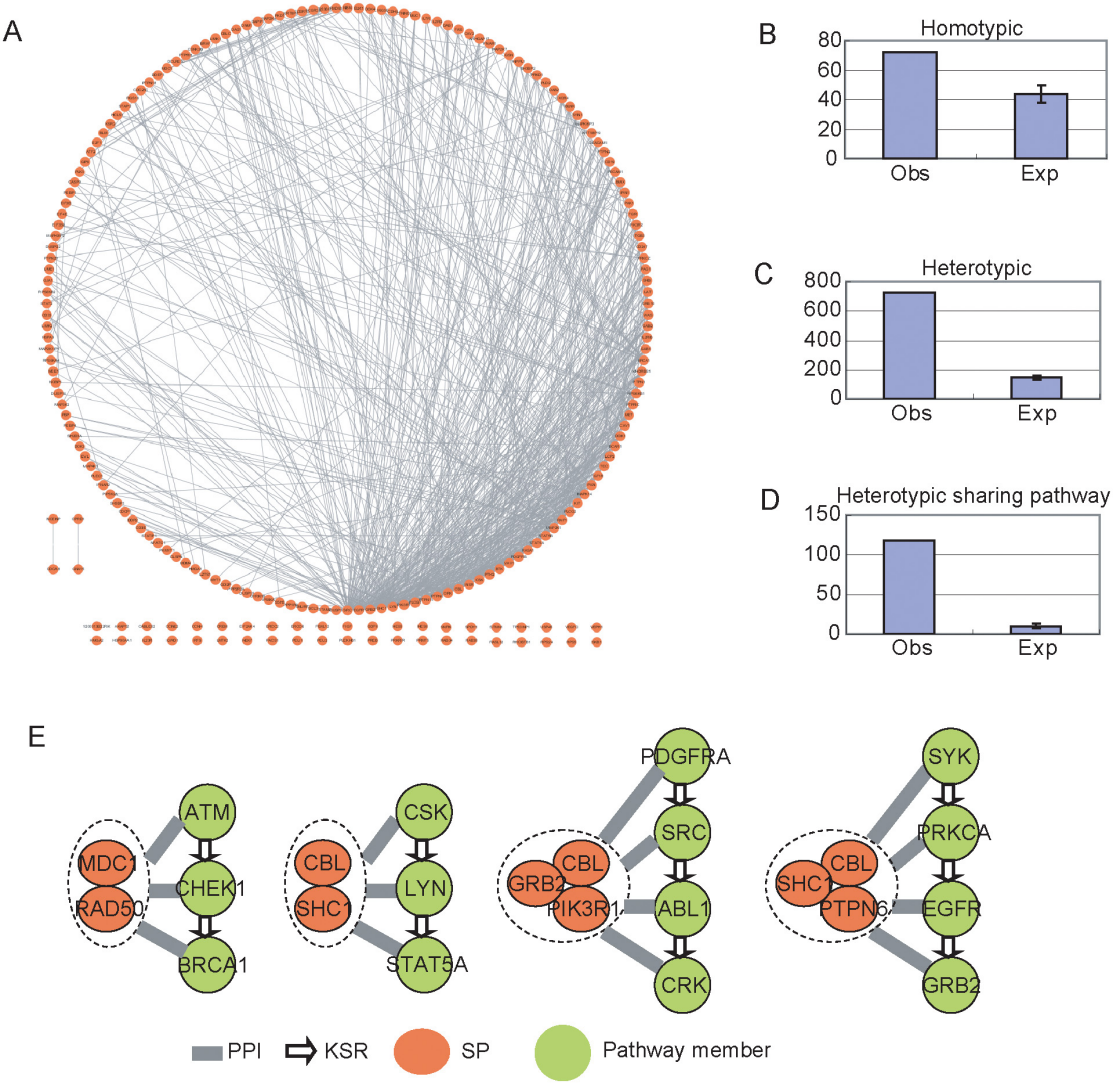
Interactions between scaffold proteins. (*A*) Overall PPI interaction networks between scaffold proteins. (*B*) Number of homotypic interactions among scaffold proteins. (*C*) Number of heterotypic interactions among scaffold proteins. (*D*) Number of heterotypic interactions that are associated with the same pathways. (*E*) Examples of scaffold protein complexes that are associated with signaling pathways. The scaffold proteins in a complex interact each other, and all of them interact with each member in the associated pathway.

Since some scaffold proteins are known to form dimers [30,31], we systematically examined the connectivity among the predicted scaffold proteins in PPI networks (Fig 6A). Among the 212 scaffold proteins, 72 of them (33.9%) have homotypic interactions, suggesting that scaffold proteins tend to form homodimers (Fig 6B). In contrast, only 20.7% of proteins (2423/11696) are found to interact with themselves for all proteins in human PPI network. The enrichment for homotypic interactions among scaffold proteins is statistically significant (*p* = 4.19×10^−6^, hypergeometric distribution).

Because it is also possible that two different scaffold proteins might form a heterodimer, we next examined the heterotypic interactions among the scaffold proteins. Among the 212 scaffold proteins, we identified total 725 PPIs (Fig 6A). As a control, we randomly selected 212 proteins with PPI degrees similar to the 212 scaffold proteins so that the effect of interactions degree was excluded. The expected number of PPIs among the 212 randomly selected proteins was only 145, suggesting that the scaffold proteins are also likely to form heterodimers (Fig 6C).

Interestingly, if we focused on the scaffold proteins associated with the same signaling pathways, we found these scaffold proteins are more likely to have heterotypic interactions. Indeed, 456 pairs of scaffold proteins share the same pathways. Among them, 118 scaffold protein pairs have direct PPIs. As a control, we randomly selected 456 pairs of protein with similar PPI degrees, and calculated the number of pairs with PPIs. We repeated the simulation 10,000 times, and the expected number of pairs with direct PPI among 456 pairs of proteins is only 10 (Fig 6D).

Our finding suggests that scaffold proteins might form scaffold protein complexes to regulate signaling pathways. For example, scaffold proteins CBL and SHC1 interact with each other; both of them are found to be associated with pathway of CSK→ LYN→STAT5A, a tyrosine kinase medicated pathway that is involved in regulation of the immune response[32]. Similarly, scaffold proteins GRB2, CBL and PIK3R1 are likely to form a scaffold complex due to the high interaction degree among them. All three proteins are predicted to be the scaffold proteins of pathway of PDGFRA→SRC→ABL1→CRK (Fig 6E), which is mediated by platelet-derived growth factor receptor and play an important role in organ development and tumor progression[33–35]. Interestingly, in this example, the scaffold protein CBL is associated with two pathways by interacting with different scaffold proteins (SHC1 vs. GRB2 and PIK3R1), suggesting that formation of scaffold protein complexes is a potential mechanism for multiplexing the function of scaffold proteins.

### Scaffold proteins themselves are likely regulated through phosphorylation

**Fig 7:**
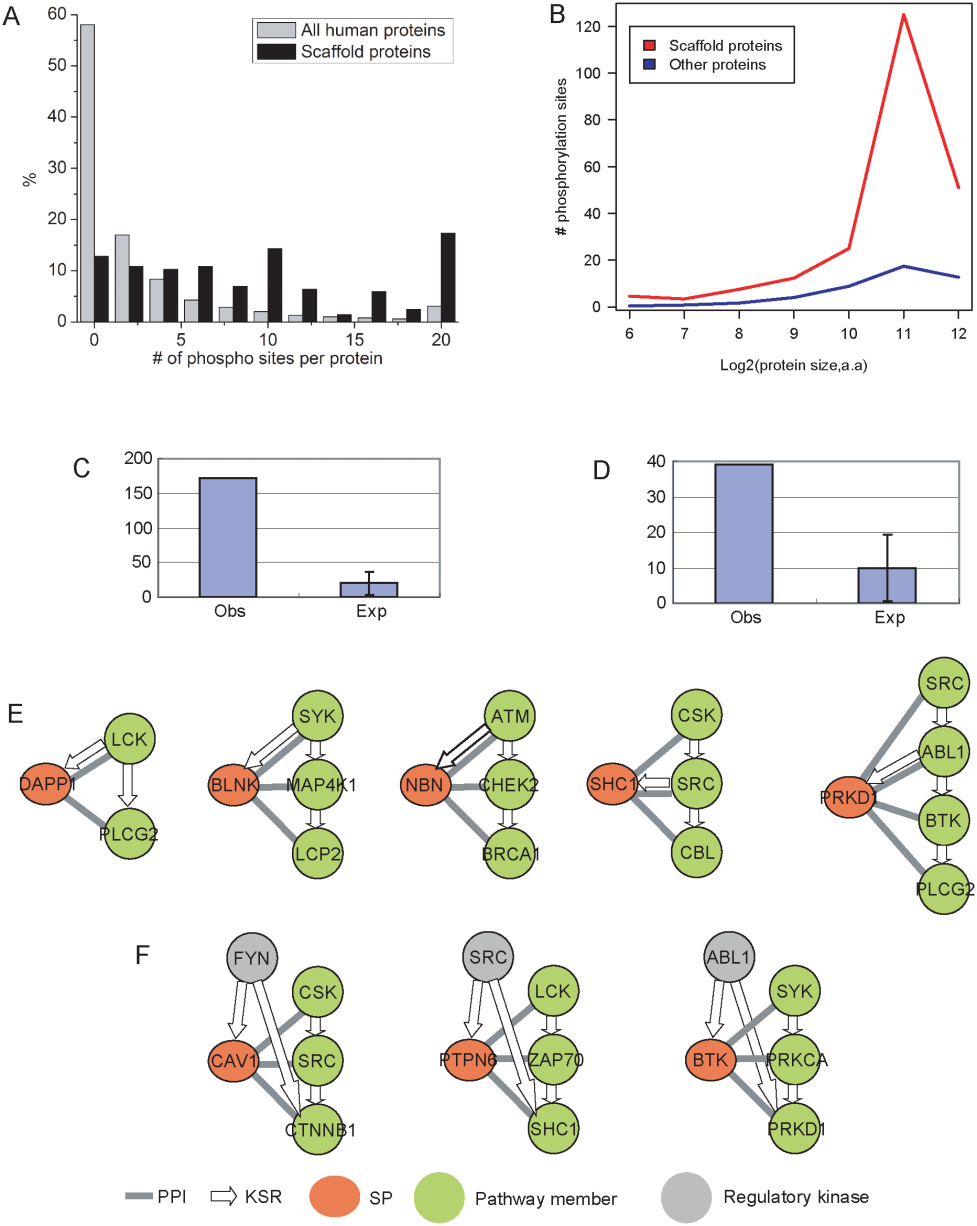
Possible regulatory mechanisms of scaffold proteins. (*A*) Distribution of MS/MS determined phosphorylation site numbers per protein. Predicted scaffold proteins have 12 sites on average. As a contrast, proteins in human proteome only have 2 sites on average. *(B)* The high number of phosphorylation sites in scaffold proteins is not due to larger protein sizes. If we compared the number of sites between scaffold proteins and the proteins with similar sizes, the similar observation was made. One possible regulatory mechanism for scaffold proteins is that one kinase member in the pathway phosphorylates the scaffold protein. The number of such cases is shown in (*C*). The examples are shown in (*E*). The second possible mechanism is that one upstream kinase phosphorylates both scaffold protein and one member in the pathways. The number of such cases is shown in (*D*). The examples are shown in (*F*).

Scaffold proteins are traditionally thought to act as “molecular glue”, bringing different protein components into proximity in a static way. It remains elusive whether and how the activity of scaffold proteins is regulated so that the scaffold proteins and the signaling pathways work in concert to respond to the environmental cue. We hypothesize that scaffold proteins within the phosphorylation network are phosphorylated themselves. To test this hypothesis, we first examined the phosphorylation sites on the predicted scaffold proteins. After collecting 70,422 known phosphorylation sites obtained from mass spectrometry experiments [3–5], we mapped these sites on the proteins. We found that the majority (98%) of predicted scaffold proteins carry at least one known phosphorylation site, and that 79% of them contain at least five known phosphorylation sites. In contrast, only 42% of proteins in the entire human proteome contain any known phosphorylation sites, and only 12.4% of them contain at least five known phosphorylation sites (Fig 7A). It is worthy to note that the property is not because of the relatively large size of scaffold proteins. If we compared the number of phosphorylation sites between scaffold proteins and the proteins with similar sizes, the scaffold proteins still have significantly more phosphorylation sites (Fig 7B). Furthermore, if we excluded kinases from the scaffold protein list, the same observation was also made (S7 Fig).

Given the possibility that scaffold proteins might be regulated through phosphorylation process, we attempted to identify the possible mechanism in which scaffold proteins and their associated pathways cooperate with each other and respond to the environment. We speculate two possible mechanisms for regulation of scaffold proteins via phosphorylation. First, we expect one kinase member in the signaling pathway phosphorylates the scaffold protein and activates it. We term such cases as intrinsic regulation. In fact, 172 cases were found in which a member in the signaling pathways phosphorylates the associated scaffold proteins, while only 20 cases were expected if we randomly selected proteins from entire human proteome as scaffold proteins (Fig 7C). For example, scaffold protein DAPP was predicted to be associated with pathway of LCK→PLCG2. Interestingly, kinase LCK in the pathway is known to phosphorylate scaffold protein DAPP [36] (Fig 7E).

The second possible mechanism is that a scaffold protein is regulated by a kinase(s) that is not a member of the scaffold protein-associated signaling pathway, which we term extrinsic regulation. Since the activity of scaffold proteins and their associated pathways are coordinated, we required that the upstream kinases regulating scaffold proteins also regulate one member in the pathway. In fact, we found 39 such cases while only 10 cases were expected (Fig 7D). For example, kinase FYN is known to phosphorylate scaffold protein CAV1, which is associated with pathway CSK→SRC→CTNNB1. In the meantime, FYN is also known to phosphorylate CTNNB1 in the pathway (Fig 7F). In summary, our study marks a promising start in identifying the regulatory mechanism of scaffold-mediated protein phosphorylation and further *in vivo* studies will determine the functional importance of our observations.

## Discussion

Signal transduction by phosphorylation is the most universal and well-studied mechanism that cells employ to mediate signal transduction, although many kinase-substrate relationships remain to be discovered. Kinase substrates have long been recognized using their known consensus sequence motifs, an amino acid sequence uniquely recognized by a particular kinase. However, with the accumulation of experimental data many kinases break this long-held rule and have been found to share similar phosphorylation motifs with other kinases, although they phosphorylate totally different sets of substrates [3]. This phenomenon has plagued the paradigm and one possible explanation is scaffold proteins. Scaffold proteins can facilitate the kinase-substrate interactions and thus, can be employed to specify and stabilize the weak and transient interactions between the members in a signaling pathway. Therefore, identification of scaffold proteins will help us make a better prediction of kinase-substrate relationships and provide us new insights into the molecular mechanisms of signal transduction. The traditional approaches to identifying the scaffold proteins are often tedious and involved in many steps [15,16]. Recently, an analysis of MAPK signaling pathways identified 10 scaffold proteins [37].

This study represents the first attempt at a large-scale projection of potential scaffold proteins. Several lines of evidences suggested the high quality of our predictions. First, comparing with other proteins, the predicted scaffold proteins showed many unique properties that are expected for scaffold proteins. For example, they are likely to contain protein domains that are known to interact with signaling proteins. Second, our protein microarray-based validations have provided a first start in validating this method. Twenty-eight protein substrates could be phosphorylated only in the presence of a predicted scaffold protein. Third, many known scaffold proteins were recovered by our predictions even if we used a very stringent cutoff of FDR = 1%. On the other hand, we are also fully aware of the limitation of our prediction. For example, we currently have only 78 known scaffold proteins. The small number will probably introduce bias in our estimation of sensitivity. Furthermore, the PPI and KSR datasets are incomplete. We believe that we will be able to improve our prediction when the known scaffold proteins, PPI and KSR datasets become more complete and accurate. More important, a better prediction could be made if we could obtain the cell type-specific PPI and KSR data in the future, because the signaling pathways are largely cell type specific.

There is one caveat of using existing protein-protein interaction (PPI) datasets for our prediction. The PPI datasets we used indeed includes both direct interactions, which were generated from yeast-two-hybrid technique, and indirect interactions, which were generated from affinity purification coupled to mass spectrometry. However, the direct interactions dominate our PPI dataset. By comparing MIPS corum database, which includes 1846 human protein complexes, only 5.9% (3243/55048) PPI interactions are from the human protein complexes. Furthermore, among the 1167 pairs of scaffold proteins and associated signaling proteins, only 88 of them (7.5%) belong to the same complexes. Therefore, we do not expect our results would be significantly affected by the inclusion of a small portion of interactions from protein complexes.

The scaffold proteins predicted in this study may only reflect a small fraction of the entire set of scaffold proteins, because we used a very stringent requirement for our prediction. In our prediction, only the proteins that interact *all* members in a pathway were considered as the candidates for the scaffold proteins, while some scaffold proteins might only interact with some members in a signaling pathway. Furthermore, we used 1% of FDR as cutoff, which is very stringent. These factors could partially explain the relatively low sensitivity of our prediction.

The systematic identification of scaffold proteins provides us the opportunity to examine the design principle of scaffold-medicated signaling pathways. Although several cases have been discovered that scaffold proteins tend to form homodimers or heterodimer [30,31], our study demonstrates that scaffold complexes are a widespread phenomenon. The statistical significance of our observation indicates that formation of complexes is a general rule of scaffold proteins. More importantly, by interacting with different partners, one scaffold protein could be involved in different signaling pathways. Therefore, formation of scaffold complexes provides a means of encoding multiplexed specificity, generating diversity and exerting additional regulatory controls in the complex signaling networks.

Despite the large body of work on scaffold proteins, little is known about whether and how scaffold proteins themselves are regulated. A few studies have showed that scaffold proteins could be regulated through phosphorylation. For example, yeast scaffold protein Ste5 was phosphorylated by Fus3, which is a member of the Ste5-associated signaling pathways [15]. However, the general regulatory mechanisms for scaffold proteins have not been extensively explored. Inspired by our finding that scaffold proteins contain many phosphorylation sites, we propose two possible mechanisms by which the scaffold proteins themselves are also subjected to phosphorylation regulation. If a scaffold protein is activated by a kinase in its associated pathway, the simultaneous activation of the scaffold protein and the associated pathway could be achieved (i.e., intrinsic regulation). Coordination can also be achieved when a scaffold protein and at least one member in its associated pathways are regulated by a kinase that is not associated with this pathway, and thereby co-activated in a concerted manner (i.e., extrinsic regulation). In both mechanisms, phosphorylation of the scaffold proteins serves as a reinforcement to ensure proper signals to be passed downstream. Future studies will further dissect the molecular mechanisms underlying the regulation of scaffold proteins. Nonetheless, our findings suggest that such regulatory mechanism might be a design principle of scaffold-mediated signal transduction.

## Materials and Methods

### Protein-protein interactions

Human protein-protein interaction (PPI) data were collected from five databases: DIP (Database of Interacting Proteins, http://dip.doe-mbi.ucla.edu), MIPS (Mammalian PPI database, http://mips.gsf.de/proj/ppi/), IntAct (ftp://ftp.ebi.ac.uk/pub/databases/intact/current/), HPRD (HPRD_Release_7_09012007, http://www.hprd.org/) and BioGRID (biogrid-all-2.0.45.tab, http://www.thebiogrid.org/downloads.php). These data were then formatted and reorganized to remove redundancies. In total, we obtained 55,048 human PPIs [17].

### KSR networks

1103 experimentally validated kinase substrate relations were collected from literature and the PhosphoELM database (phosphor.elm.eu.org) [3].

### Computation of PPI distance

The PPI distances of a protein pair is defined as the shortest distance of the protein pair in the PPI network, and can be computed using Breadth-First Search (BFS) algorithm [38]. We took each protein with PPI information as a root, and defined it as the first level of a tree. We then extended the root to take all its neighbors as the nodes at the second level of the tree. We next took the neighbors of all nodes at second level as the nodes at the third level of the tree, and all nodes that had appeared in previous levels would be deleted in this level. We repeated this procedure till no further level could be added to the tree. This resulted in the PPI distances between root node and all other nodes in the tree being the difference of their levels. For example, the PPI distance between root node (first level) and a node at the fourth level is 3. This allowed us to obtain the shortest distance of each protein pair.

### Identification of phosphorylation-related scaffold proteins

We first extracted all pathways from a KSR network. We took each kinase as root, and extended its substrates using a Depth-First Search (DFS) algorithm [39]. Each path starting from a root in the tree represents a possible phosphorylation pathway. Here, we require the path must start from a root node, but does not need to end at a leaf node. The minimum length of a pathway was set as 2. To speed up the program, only KSR with PPI distance of one or two are considered to build the pathways since KSR with PPI distance larger than two don’t share neighbors in PPI network thus it is impossible for them to have a related scaffold protein. We also included the continuous sub-pathways of long pathways because the longer pathways may not have corresponding scaffold proteins, while its continuous substrings do. By doing it this way, we can list all possible pathways and remove any redundancies.

For each possible pathway, we checked whether all protein in the pathway had a common interacting partner in PPI network. If so, the common interacting partner is predicted as candidate scaffold protein related to that pathway.

### False discovery rate (FDR) control

FDR is a statistical method to control the false positive rate in predicted result, which is especially useful in multiple-hypothesis testing to correct for multiple comparisons [40]. In practice, FDR can be defined as the expected false positive rate. Supposing there are n independent tests, each test contains m_i_ predicted results with FDR q_i_ (q_i_ ≤q* for i = 1,…,n), then the integral FDR q satisfies the following formula,

$$q=\frac{\sum_{i=1}^{n}m_{i}\times q_{i}}{\sum_{i=1}^{n}m_{i}}\leq \frac{\sum_{i=1}^{n}m_{i}\times q^{*}}{\sum_{i=1}^{n}m_{i}}=q^{*}\times \frac{\sum_{i=1}^{n}m_{i}}{\sum_{i=1}^{n}m_{i}}=q^{*}$$

In our case, each candidate scaffold protein corresponds to one independent test, thus we can control the integral FDR by controlling the FDR of each individual scaffold protein. Suppose *l* is the pathway length cutoff and SP is a scaffold protein, SP corresponds to N pathways with length ≧*l* based on real PPI data, and corresponds to M pathways with length ≧*l* based on random PPI data, then the FDR of SP as well as its related pathways under pathway length cutoff *l* can be estimated as M/N.

The random PPI data was produced by the random shuffle of real human PPI data. We randomly selected two human PPI pairs, such as A-B and C-D, and then exchange their partners to create two new pairs, A-D and B-C. These two pairs will replace A-B and C-D if both of them are not included in real human PPI data. We repeated this procedure as many times as that of the total number of PPI pairs to create the random PPI data and each pair has been shuffled about two times on average. The final random PPI data also contain exactly 55,048 PPI pairs. This kind of shuffle breaks the biological relationship between a protein and its PPI partners, but does not change its PPI degree or the number of its PPI partners, thus keeps its major characters of statistics. Based on the shuffled random PPI data, we can compute the pathways related to a candidate scaffold protein. For accuracy, we created 1000 random PPI data and use them to calculate the average length cutoff under false discovery rate of 0.01.

### Extracting known scaffold proteins

We use “scaffold protein” as keyword to search papers in google scholar and pubmed to find all papers containing this keyword. We then manually collected the known scaffold proteins (S2 Table).

### Protein purification

Proteins for the microarrays were purified, printed, and analyzed as described previously (Jeong et al, 2012). Kinases and scaffolds ORFs were expressed as GST-fusion proteins in yeast. Cultures (50 mL) were grown at 30°C to OD_600_ 1.0–1.2 and induced with 2% galactose for 4–6 hours. Harvested cells were lysed with glass beads in lysis buffer (100 mMTris-HCl [pH 7.4], 100 mMNaCl, 1 mM EGTA, 0.1% 2-mercaptoethanol, 0.5 mM PMSF, 0.1% Triton X-100, protease inhibitor cocktail [Roche], and phosphatase inhibitor cocktails 2 and 3 [Sigma]). GST-proteins were bound to glutathione beads (GE healthcare) for 40 minutes at 4°C and washed 3 times with Wash Buffer I (50 mMTris-HCl [pH 7.4], 500 mMNaCl, 1 mM EGTA, 10% glycerol, 0.1% Triton X-100, 0.1% 2-mercaptoethanol, and 0.5 mM PMSF) and 3 times with Wash Buffer II (50 mM HEPES [pH 7.4], 100 mMNaCl, 1 mM EGTA, 10% glycerol, 0.1% 2-mercaptoethanol, and 0.5 mM PMSF) before 2 30 minute elutions in elution buffer (100 mMTris-HCl [pH 8.0], 100 mMNaCl, 10 mM MgCl_2_, 30 mM glutathione, and 20% glycerol). Eluate was collected and concentrations were determined through BSA standard.

### Dot blot assay

Purified kinase activity was assessed using a simple dot blot assay by incubating each kinase with a generic substrate mix in the presence of ^32^P-γ-ATP. 2 μL purified kinases were mixed with 1 μL substrate mix (1:1:1 casein:MBP:Histone H3 100 ng/μL dissolved in TBS) and 2μL 2.5x reaction buffer (90 mMTris-HCl, pH 7.5, 180 mMNaCl, 9 mM MgCl_2_, 0.9 mM MnCl_2_, 0.9 mM DTT, 9μM cold ATP, 2.5 mM EGTA, 20 mM HEPES-KOH, pH 7.5, 0.9 mMNaF, 0.9 mM Na_3_VO_4_, and 5.954E-05 mM^32^P-γ-ATP [Perkin Elmer; 0.2 μL/5.6μL reaction mix]) and incubated at 30°C for 30 minutes. Reactions were quenched by spotting entire mix onto nitrocellulose paper and drying for 15 minutes. Membrane was then washed 3 times for 10 minutes with PBS and dried again for 15 minutes. Blots were exposed to film overnight.

### Protein microarray assay

In order to assess whether predicted scaffolds could alter kinase substrate specificity, protein microarray assays were performed. In these assays, the protein microarrays were treated with purified, active kinase both in the absence and presence of predicted scaffold. Microarrays were briefly dipped in TBS to remove excess glycerol from printing procedure before blocking in 3 mL of blocking buffer (3% BSA in TBST) for 1 hour. Arrays were washed 3 times in TBST before the addition of 125 uL of kinase buffer containing 3:1 scaffold:kinase in kinase buffer (50 mMTris-HCl [pH 7.5], 100 mMNaCl, 10mM MgCl_2_, 1 mM MnCl_2_, 1 mM DTT, 1 mM EGTA, 25 mM HEPES-KOH [pH 7.5], 1 mM NaVO_4_, 1 mMNaF, 0.1% NP-40, 0.0000556 mM^33^P-γ-ATP [Perkin Elmer; 2 μL/array]). Arrays were placed in a humidity chamber and incubated for 30 minutes at 30°C. Following the reaction, arrays were quickly immersed in two separate beakers of TBST and washed 3 times in TBST for 10 minutes followed by 3 washes in 0.5% SDS for 10 minutes. Arrays were then quickly dunked in water heated to 37°C and dried by centrifugation before being arranged in a standard film cassette and exposed to film (Kodak BioMax MR) for 30 days at -80°C. After 30 days, the film was developed and scanned before analysis with GenePix software.

## Supporting Information

S1 Fig Network of predicted scaffold proteins and proteins in their associated pathways. Scaffold proteins were colored as red and proteins in pathways were colored as green. Scaffold proteins were colored as blue if they were also proteins in pathways. Dashed arrows represent the relationships from scaffold proteins to proteins in the related pathways. Note that the relationships represented by dashed arrows are different to the solid arrows in the manuscript, which represent the KSRs.
(TIFF)

S2 Fig The PPI degreee distribution for predicted scaffold proteins. (TIFF)

S3 Fig The percentage of scaffold proteins of human proteins with PPI degrees. The larger the PPI degrees, the more possible a protein to be a scaffold protein, but not all high-degree proteins are predicted as scaffold proteins. For example, only 20% of proteins with degree greater than 140 are predicted as scaffold proteins.
(TIFF)

S4 Fig Coomassie stain of scaffold proteins and kinases shows high quality of purification. Dot blot using generic substrate mix indicates that kinases are very active against generic substrates.
(TIFF)

S5 Fig Comparison of protein sizes between scaffold proteins and other proteins with similar PPI degree. (TIFF)

S6 Fig Correlation coefficient of gene expression between scaffold proteins and their associated pathway members (red line). The background distribution (blue line) represents the same correlation coefficients between two randomly selected genes. The difference between the two distributions is statistically significant.
(TIFF)

S7 Fig Comparison of the numbers of phosphorylation sites between non-kinase scaffold proteins and other human proteins with similar protein sizes. (TIFF)

S1 Table Predicted scaffold proteins and their associated pathways. (XLS)

S2 Table List of 78 known scaffold proteins. (Proteins recovered by our prediction are in bold; the corresponding references of the scaffold proteins are also listed.)
(DOCX)

S3 Table Validation hit list of predicted scaffold proteins. (DOCX)

S4 Table List of *PIN1*and *ATF2* meditated phosphorylation events. Each array was manually aligned and scored. Final hits that were identified were replicated, not present in control arrays, and had F/B ratios >1.5. Proteins in bold were hit with both *ATF2* and *PIN1*.
(DOCX)
