# Supplementary figures and images for: Systematic Prediction of Scaffold Proteins Reveals New Design Principles in Scaffold-Mediated Signal Transduction
Source: PLoS Comput Biol. 2015 Sep 22;11(9):e1004508. doi: 10.1371/journal.pcbi.1004508 (PMC4578958; doi:10.1371/journal.pcbi.1004508)

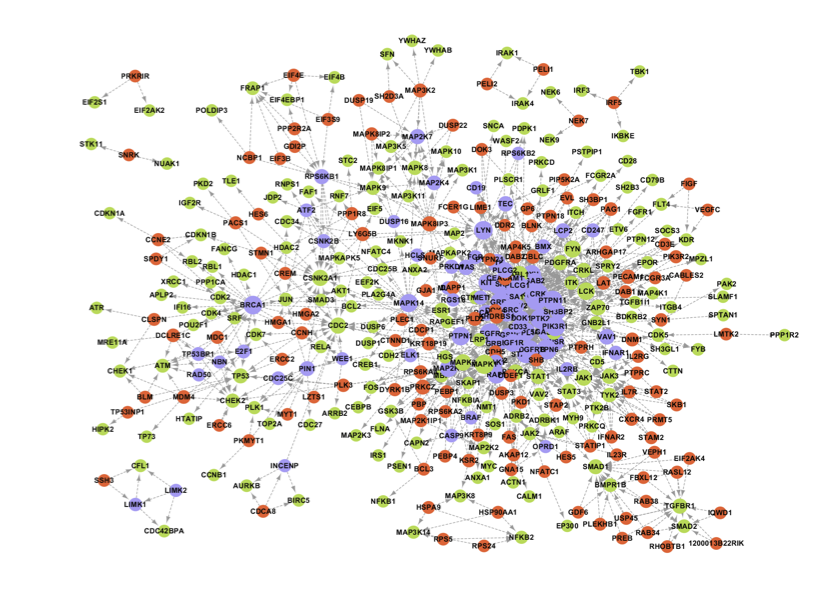

Supplement: S1 Fig — Scaffold proteins were colored as red and proteins in pathways were colored as green. Scaffold proteins were colored as blue if they were also proteins in pathways. Dashed arrows represent the relationships from scaffold proteins to proteins in the related pathways. Note that the relationships represented by dashed arrows are different to the solid arrows in the manuscript, which represent the KSRs. (TIFF) [file pcbi.1004508.s001.tiff]

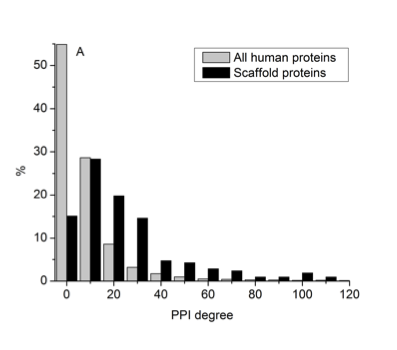

Supplement: S2 Fig — (TIFF) [file pcbi.1004508.s002.tiff]

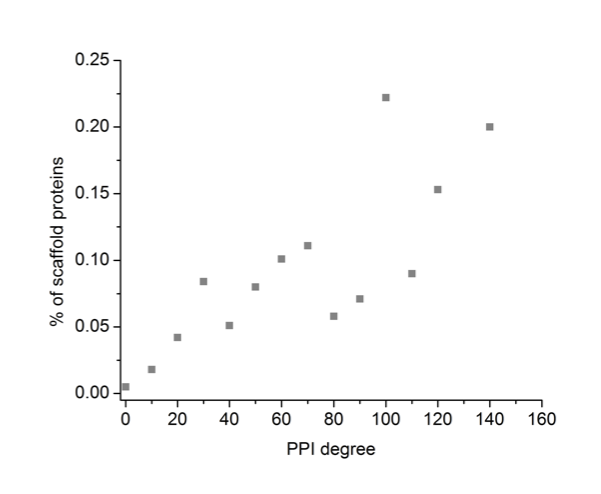

Supplement: S3 Fig — The larger the PPI degrees, the more possible a protein to be a scaffold protein, but not all high-degree proteins are predicted as scaffold proteins. For example, only 20% of proteins with degree greater than 140 are predicted as scaffold proteins. (TIFF) [file pcbi.1004508.s003.tiff]

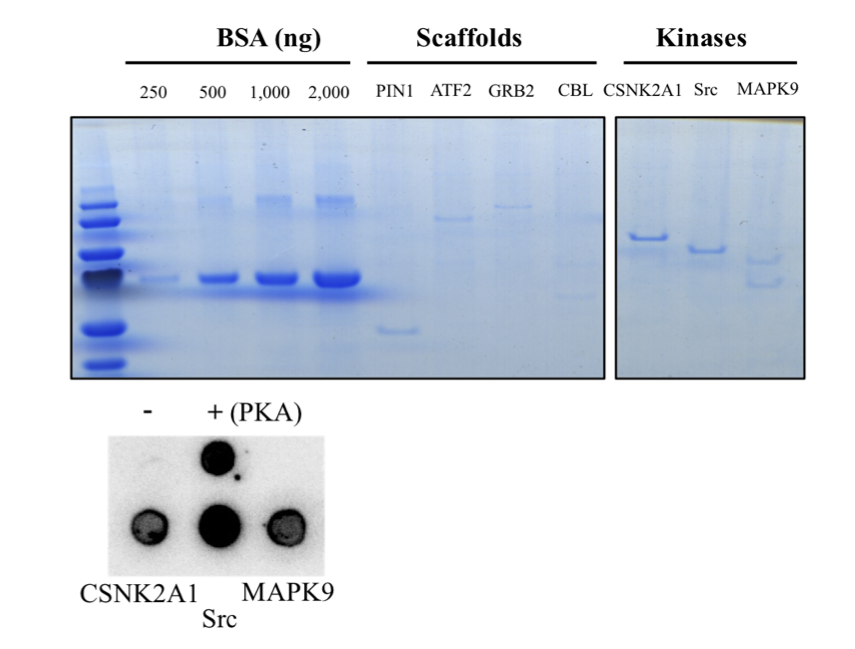

Supplement: S4 Fig — Dot blot using generic substrate mix indicates that kinases are very active against generic substrates. (TIFF) [file pcbi.1004508.s004.tiff]

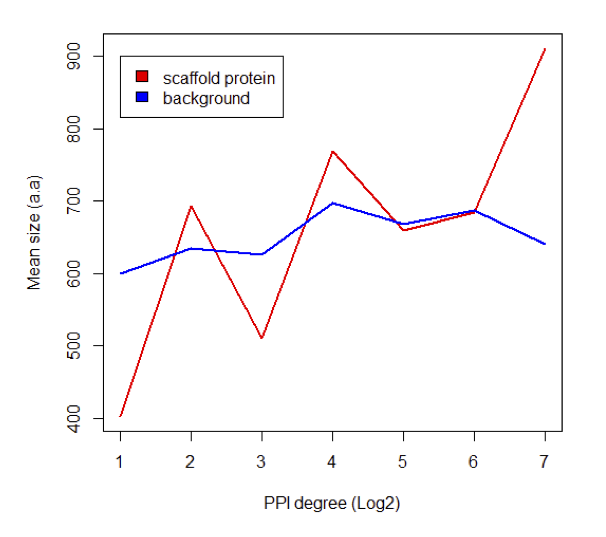

Supplement: S5 Fig — (TIFF) [file pcbi.1004508.s005.tiff]

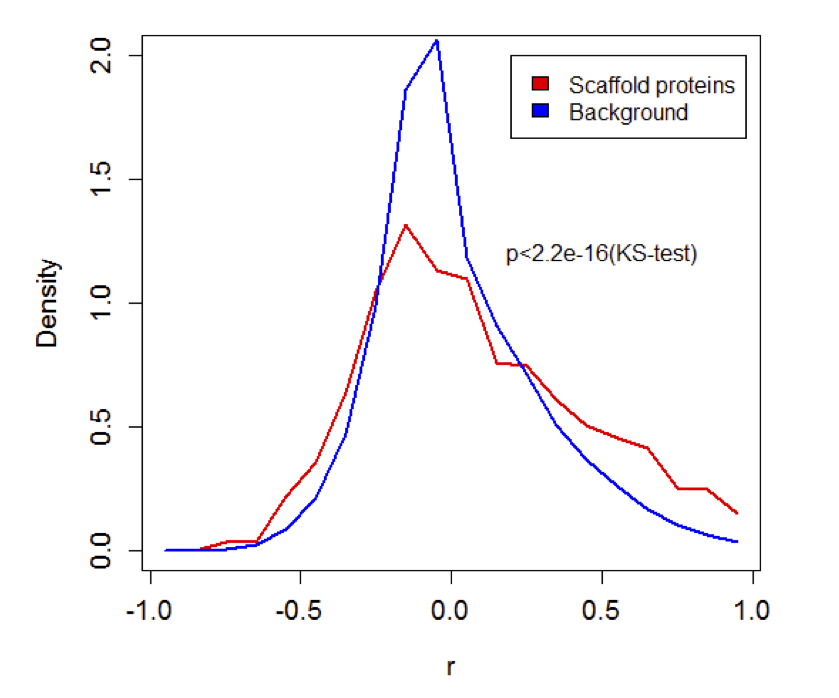

Supplement: S6 Fig — The background distribution (blue line) represents the same correlation coefficients between two randomly selected genes. The difference between the two distributions is statistically significant. (TIFF) [file pcbi.1004508.s006.tiff]

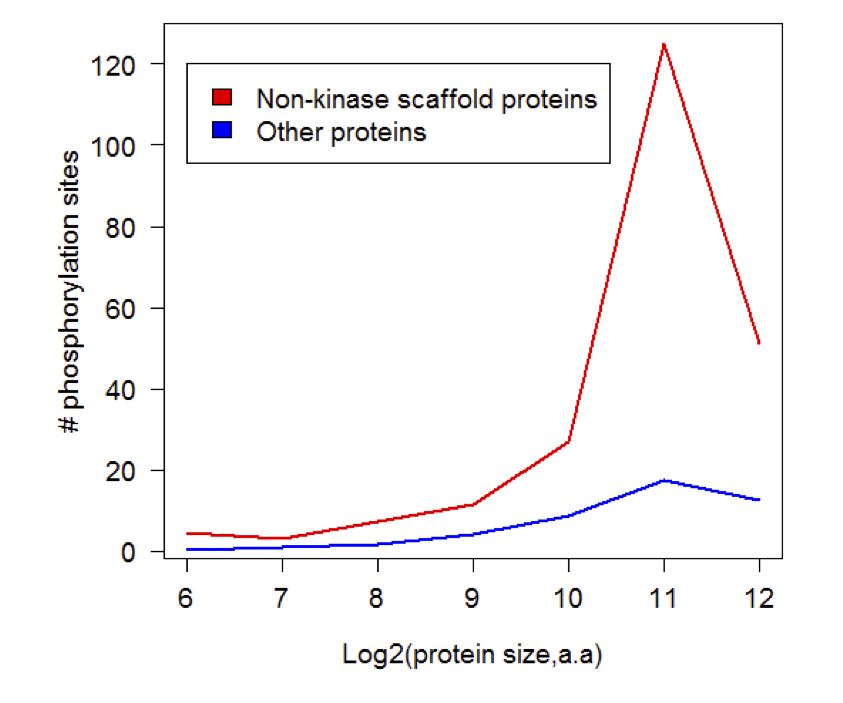

Supplement: S7 Fig — (TIFF) [file pcbi.1004508.s007.tiff]
