# Supplementary material for: Systematic Prediction of Scaffold Proteins Reveals New Design Principles in Scaffold-Mediated Signal Transduction
Source: PLoS Comput Biol. 2015 Sep 22;11(9):e1004508. doi: 10.1371/journal.pcbi.1004508 (PMC4578958; doi:10.1371/journal.pcbi.1004508)
Supplement: S2 Table — (Proteins recovered by our prediction are in bold; the corresponding references of the scaffold proteins are also listed.) (DOCX) [file pcbi.1004508.s009.docx]

**S2 Table.** List of 78 known scaffold proteins (proteins recovered by our prediction are in bold; the corresponding references of the scaffold proteins are also listed)**.**

| Official Symbol | Official Symbol | Official Symbol | Official Symbol |
| --- | --- | --- | --- |
| ABI3([*1*](#_ENREF_1)) | EIF4G1([*2*](#_ENREF_2)*,* [*3*](#_ENREF_3)) | **MAP2K1IP1**([*4*](#_ENREF_4)) | RIMS1([*5*](#_ENREF_5)) |
| AHNAK([*6*](#_ENREF_6)*,* [*7*](#_ENREF_7)) | EIF4G2([*2*](#_ENREF_2)*,* [*3*](#_ENREF_3)) | **MAP2K4**([*4*](#_ENREF_4)) | SH3RF1([*8*](#_ENREF_8)) |
| **AKAP12**([*1*](#_ENREF_1)*,* [*9*](#_ENREF_9)) | EIF4G3([*2*](#_ENREF_2)*,* [*3*](#_ENREF_3)) | MAP3K1([*7*](#_ENREF_7)) | **SHC1**([*1*](#_ENREF_1)) |
| AKAP5([*10*](#_ENREF_10)) | ERBB2([*11*](#_ENREF_11)) | MAPK8IP1([*11*](#_ENREF_11)) | SHOC2([*12*](#_ENREF_12)) |
| AKAP9([*13*](#_ENREF_13)) | FLNA([*14*](#_ENREF_14)) | **MAPK8IP2**([*15*](#_ENREF_15)) | SLC9A3R1([*14*](#_ENREF_14)) |
| ARRB1([*16*](#_ENREF_16)) | FRS2([*11*](#_ENREF_11)) | **MAPK8IP3**([*17*](#_ENREF_17)) | SLC9A3R2([*14*](#_ENREF_14)) |
| ARRB2([*16*](#_ENREF_16)) | **GAB1**([*18*](#_ENREF_18)) | MPDZ([*14*](#_ENREF_14)) | SNTA1([*19*](#_ENREF_19)) |
| AXIN1([*1*](#_ENREF_1)*,* [*20*](#_ENREF_20)) | **GAB2**([*21*](#_ENREF_21)) | PARD3([*11*](#_ENREF_11)) | SPAG9([*22*](#_ENREF_22)) |
| BCL10([*7*](#_ENREF_7)) | GNB2L1([*23*](#_ENREF_23)) | PDZK1([*24*](#_ENREF_24)) | SQSTM1([*25*](#_ENREF_25)) |
| **CAV1**([*1*](#_ENREF_1)*,* [*26*](#_ENREF_26)) | **GRB2**([*1*](#_ENREF_1)) | **PELI1**([*7*](#_ENREF_7)) | STRN([*27*](#_ENREF_27)) |
| **CBL**([*1*](#_ENREF_1)) | IKBKAP([*28*](#_ENREF_28)) | **PELI2**([*7*](#_ENREF_7)) | TANC1([*29*](#_ENREF_29)) |
| CD2([*30*](#_ENREF_30)) | INADL([*14*](#_ENREF_14)) | PELI3([*7*](#_ENREF_7)) | TJP1([*31*](#_ENREF_31)) |
| CD2AP([*32*](#_ENREF_32)) | IQGAP1([*33*](#_ENREF_33)) | PICK1([*34*](#_ENREF_34)) | TRADD([*11*](#_ENREF_11)) |
| CNKSR1([*35*](#_ENREF_35)) | IRS1([*1*](#_ENREF_1)) | **PPP1R8**([*36*](#_ENREF_36)) | TRPC4AP([*31*](#_ENREF_31)) |
| **CRK**([*1*](#_ENREF_1)) | JAK2([*14*](#_ENREF_14)) | PPP1R9B([*7*](#_ENREF_7)) | WASF1([*37*](#_ENREF_37)) |
| DLG1([*7*](#_ENREF_7)*,* [*38*](#_ENREF_38)) | KSR1([*7*](#_ENREF_7)) | PPP2R5A([*1*](#_ENREF_1)) | XRCC1([*39*](#_ENREF_39)) |
| DLG4([*14*](#_ENREF_14)*,* [*40*](#_ENREF_40)) | **KSR2**([*7*](#_ENREF_7)) | PRKCABP([*14*](#_ENREF_14)) | YWHAG([*14*](#_ENREF_14)) |
| **DUSP19**([*41*](#_ENREF_41)) | **LCP2**([*11*](#_ENREF_11)) | PTPN12([*42*](#_ENREF_42)) | YWHAZ([*43*](#_ENREF_43)) |
| DVL1([*1*](#_ENREF_1)) | LGALS3([*44*](#_ENREF_44)) | RANBP9([*45*](#_ENREF_45)) |  |
| DVL2([*1*](#_ENREF_1)) | MAGI2([*46*](#_ENREF_46)) | RAPSN([*47*](#_ENREF_47)) |  |

**Additional References**

1. M. J. Berridge, Cell Signaling Biology (module 6). *doi:10.1042/csb0001001*, (2011).

2. T. M. Hinton, M. J. Coldwell, G. A. Carpenter, S. J. Morley, V. M. Pain, Functional analysis of individual binding activities of the scaffold protein eIF4G. *J Biol Chem* **282**, 1695-1708 (2007).

3. S. Pyronnet, H. Imataka, A. C. Gingras, R. Fukunaga, T. Hunter, N. Sonenberg, Human eukaryotic translation initiation factor 4G (eIF4G) recruits mnk1 to phosphorylate eIF4E. *Embo J* **18**, 270-279 (1999).

4. T. P. Garrington, G. L. Johnson, Organization and regulation of mitogen-activated protein kinase signaling pathways. *Curr Opin Cell Biol* **11**, 211-218 (1999).

5. R. Khanna, Q. Li, L. Sun, T. J. Collins, E. F. Stanley, N type Ca2+ channels and RIM scaffold protein covary at the presynaptic transmitter release face but are components of independent protein complexes. *Neuroscience* **140**, 1201-1208 (2006).

6. D. Matza, A. Badou, K. S. Kobayashi, K. Goldsmith-Pestana, Y. Masuda, A. Komuro, D. McMahon-Pratt, V. T. Marchesi, R. A. Flavell, A scaffold protein, AHNAK1, is required for calcium signaling during T cell activation. *Immunity* **28**, 64-74 (2008).

7. A. S. Shaw, E. L. Filbert, Scaffold proteins and immune-cell signalling. *Nat Rev Immunol* **9**, 47-56 (2009).

8. N. Tapon, K. Nagata, N. Lamarche, A. Hall, A new rac target POSH is an SH3-containing scaffold protein involved in the JNK and NF-kappaB signalling pathways. *Embo J* **17**, 1395-1404 (1998).

9. J. B. Nauert, T. M. Klauck, L. K. Langeberg, J. D. Scott, Gravin, an autoantigen recognized by serum from myasthenia gravis patients, is a kinase scaffold protein. *Curr Biol* **7**, 52-62 (1997).

10. T. M. Klauck, M. C. Faux, K. Labudda, L. K. Langeberg, S. Jaken, J. D. Scott, Coordination of three signaling enzymes by AKAP79, a mammalian scaffold protein. *Science (New York, N.Y* **271**, 1589-1592 (1996).

11. T. Pawson, P. Nash, Protein-protein interactions define specificity in signal transduction. *Genes & development* **14**, 1027-1047 (2000).

12. R. Matsunaga-Udagawa, Y. Fujita, S. Yoshiki, K. Terai, Y. Kamioka, E. Kiyokawa, K. Yugi, K. Aoki, M. Matsuda, The scaffold protein Shoc2/SUR-8 accelerates the interaction of Ras and Raf. *J Biol Chem* **285**, 7818-7826 (2010).

13. M. Takahashi, H. Shibata, M. Shimakawa, M. Miyamoto, H. Mukai, Y. Ono, Characterization of a novel giant scaffolding protein, CG-NAP, that anchors multiple signaling enzymes to centrosome and the golgi apparatus. *J Biol Chem* **274**, 17267-17274 (1999).

14. R. A. Hall, R. J. Lefkowitz, Regulation of G protein-coupled receptor signaling by scaffold proteins. *Circ Res* **91**, 672-680 (2002).

15. N. Kelkar, C. L. Standen, R. J. Davis, Role of the JIP4 scaffold protein in the regulation of mitogen-activated protein kinase signaling pathways. *Mol Cell Biol* **25**, 2733-2743 (2005).

16. V. Bryja, D. Gradl, A. Schambony, E. Arenas, G. Schulte, Beta-arrestin is a necessary component of Wnt/beta-catenin signaling in vitro and in vivo. *Proceedings of the National Academy of Sciences of the United States of America* **104**, 6690-6695 (2007).

17. N. Kelkar, M. H. Delmotte, C. R. Weston, T. Barrett, B. J. Sheppard, R. A. Flavell, R. J. Davis, Morphogenesis of the telencephalic commissure requires scaffold protein JNK-interacting protein 3 (JIP3). *Proceedings of the National Academy of Sciences of the United States of America* **100**, 9843-9848 (2003).

18. A. Kiyatkin, E. Aksamitiene, N. I. Markevich, N. M. Borisov, J. B. Hoek, B. N. Kholodenko, Scaffolding protein Grb2-associated binder 1 sustains epidermal growth factor-induced mitogenic and survival signaling by multiple positive feedback loops. *J Biol Chem* **281**, 19925-19938 (2006).

19. A. D. Bragg, M. Amiry-Moghaddam, O. P. Ottersen, M. E. Adams, S. C. Froehner, Assembly of a perivascular astrocyte protein scaffold at the mammalian blood-brain barrier is dependent on alpha-syntrophin. *Glia* **53**, 879-890 (2006).

20. W. Liu, H. Rui, J. Wang, S. Lin, Y. He, M. Chen, Q. Li, Z. Ye, S. Zhang, S. C. Chan, Y. G. Chen, J. Han, S. C. Lin, Axin is a scaffold protein in TGF-beta signaling that promotes degradation of Smad7 by Arkadia. *Embo J* **25**, 1646-1658 (2006).

21. Y. Liu, B. Jenkins, J. L. Shin, L. R. Rohrschneider, Scaffolding protein Gab2 mediates differentiation signaling downstream of Fms receptor tyrosine kinase. *Mol Cell Biol* **21**, 3047-3056 (2001).

22. G. Takaesu, J. S. Kang, G. U. Bae, M. J. Yi, C. M. Lee, E. P. Reddy, R. S. Krauss, Activation of p38alpha/beta MAPK in myogenesis via binding of the scaffold protein JLP to the cell surface protein Cdo. *J Cell Biol* **175**, 383-388 (2006).

23. S. J. Yarwood, M. R. Steele, G. Scotland, M. D. Houslay, G. B. Bolger, The RACK1 signaling scaffold protein selectively interacts with the cAMP-specific phosphodiesterase PDE4D5 isoform. *J Biol Chem* **274**, 14909-14917 (1999).

24. E. K. Malmberg, C. X. Andersson, M. Gentzsch, J. H. Chen, A. Mengos, L. Cui, G. C. Hansson, J. R. Riordan, Bcr (breakpoint cluster region) protein binds to PDZ-domains of scaffold protein PDZK1 and vesicle coat protein Mint3. *J Cell Sci* **117**, 5535-5541 (2004).

25. J. Moscat, M. T. Diaz-Meco, M. W. Wooten, Signal integration and diversification through the p62 scaffold protein. *Trends Biochem Sci* **32**, 95-100 (2007).

26. S. Li, J. Couet, M. P. Lisanti, Src tyrosine kinases, Galpha subunits, and H-Ras share a common membrane-anchored scaffolding protein, caveolin. Caveolin binding negatively regulates the auto-activation of Src tyrosine kinases. *J Biol Chem* **271**, 29182-29190 (1996).

27. S. Gaillard, M. Bartoli, F. Castets, A. Monneron, Striatin, a calmodulin-dependent scaffolding protein, directly binds caveolin-1. *FEBS letters* **508**, 49-52 (2001).

28. L. Cohen, W. J. Henzel, P. A. Baeuerle, IKAP is a scaffold protein of the IkappaB kinase complex. *Nature* **395**, 292-296 (1998).

29. T. Suzuki, W. Li, J. P. Zhang, Q. B. Tian, H. Sakagami, N. Usuda, H. Kondo, T. Fujii, S. Endo, A novel scaffold protein, TANC, possibly a rat homolog of Drosophila rolling pebbles (rols), forms a multiprotein complex with various postsynaptic density proteins. *Eur J Neurosci* **21**, 339-350 (2005).

30. Y. Ye, H. W. Lee, W. Yang, S. J. Shealy, A. L. Wilkins, Z. R. Liu, I. Torshin, R. Harrison, R. Wohlhueter, J. J. Yang, Metal binding affinity and structural properties of an isolated EF-loop in a scaffold protein. *Protein Eng* **14**, 1001-1013 (2001).

31. S. M. Soond, J. L. Terry, J. D. Colbert, D. W. Riches, TRUSS, a novel tumor necrosis factor receptor 1 scaffolding protein that mediates activation of the transcription factor NF-kappaB. *Mol Cell Biol* **23**, 8334-8344 (2003).

32. D. K. Lynch, S. C. Winata, R. J. Lyons, W. E. Hughes, G. M. Lehrbach, V. Wasinger, G. Corthals, S. Cordwell, R. J. Daly, A Cortactin-CD2-associated protein (CD2AP) complex provides a novel link between epidermal growth factor receptor endocytosis and the actin cytoskeleton. *J Biol Chem* **278**, 21805-21813 (2003).

33. H. Nakamura, K. Fujita, H. Nakagawa, F. Kishi, A. Takeuchi, I. Aute, H. Kato, Expression pattern of the scaffold protein IQGAP1 in lung cancer. *Oncol Rep* **13**, 427-431 (2005).

34. N. Reymond, S. Garrido-Urbani, J. P. Borg, P. Dubreuil, M. Lopez, PICK-1: a scaffold protein that interacts with Nectins and JAMs at cell junctions. *FEBS letters* **579**, 2243-2249 (2005).

35. S. Rabizadeh, R. J. Xavier, K. Ishiguro, J. Bernabeortiz, M. Lopez-Ilasaca, A. Khokhlatchev, P. Mollahan, G. P. Pfeifer, J. Avruch, B. Seed, The scaffold protein CNK1 interacts with the tumor suppressor RASSF1A and augments RASSF1A-induced cell death. *J Biol Chem* **279**, 29247-29254 (2004).

36. A. Van Eynde, M. Nuytten, M. Dewerchin, L. Schoonjans, S. Keppens, M. Beullens, L. Moons, P. Carmeliet, W. Stalmans, M. Bollen, The nuclear scaffold protein NIPP1 is essential for early embryonic development and cell proliferation. *Mol Cell Biol* **24**, 5863-5874 (2004).

37. R. S. Westphal, S. H. Soderling, N. M. Alto, L. K. Langeberg, J. D. Scott, Scar/WAVE-1, a Wiskott-Aldrich syndrome protein, assembles an actin-associated multi-kinase scaffold. *Embo J* **19**, 4589-4600 (2000).

38. J. L. Round, L. A. Humphries, T. Tomassian, P. Mittelstadt, M. Zhang, M. C. Miceli, Scaffold protein Dlgh1 coordinates alternative p38 kinase activation, directing T cell receptor signals toward NFAT but not NF-kappaB transcription factors. *Nat Immunol* **8**, 154-161 (2007).

39. Y. Kubota, R. A. Nash, A. Klungland, P. Schar, D. E. Barnes, T. Lindahl, Reconstitution of DNA base excision-repair with purified human proteins: interaction between DNA polymerase beta and the XRCC1 protein. *Embo J* **15**, 6662-6670 (1996).

40. B. Cubelos, I. M. Gonzalez-Gonzalez, C. Gimenez, F. Zafra, The scaffolding protein PSD-95 interacts with the glycine transporter GLYT1 and impairs its internalization. *J Neurochem* **95**, 1047-1058 (2005).

41. T. Zama, R. Aoki, T. Kamimoto, K. Inoue, Y. Ikeda, M. Hagiwara, Scaffold role of a mitogen-activated protein kinase phosphatase, SKRP1, for the JNK signaling pathway. *J Biol Chem* **277**, 23919-23926 (2002).

42. D. Davidson, A. Veillette, PTP-PEST, a scaffold protein tyrosine phosphatase, negatively regulates lymphocyte activation by targeting a unique set of substrates. *Embo J* **20**, 3414-3426 (2001).

43. J. Frasor, E. C. Chang, B. Komm, C. Y. Lin, V. B. Vega, E. T. Liu, L. D. Miller, J. Smeds, J. Bergh, B. S. Katzenellenbogen, Gene expression preferentially regulated by tamoxifen in breast cancer cells and correlations with clinical outcome. *Cancer Res* **66**, 7334-7340 (2006).

44. R. Shalom-Feuerstein, S. J. Plowman, B. Rotblat, N. Ariotti, T. Tian, J. F. Hancock, Y. Kloog, K-ras nanoclustering is subverted by overexpression of the scaffold protein galectin-3. *Cancer Res* **68**, 6608-6616 (2008).

45. L. C. Murrin, J. N. Talbot, RanBPM, a scaffolding protein in the immune and nervous systems. *J Neuroimmune Pharmacol* **2**, 290-295 (2007).

46. C. R. Marshall, E. J. Young, A. M. Pani, M. L. Freckmann, Y. Lacassie, C. Howald, K. K. Fitzgerald, M. Peippo, C. A. Morris, K. Shane, M. Priolo, M. Morimoto, I. Kondo, E. Manguoglu, S. Berker-Karauzum, P. Edery, H. H. Hobart, C. B. Mervis, O. Zuffardi, A. Reymond, P. Kaplan, M. Tassabehji, R. G. Gregg, S. W. Scherer, L. R. Osborne, Infantile spasms is associated with deletion of the MAGI2 gene on chromosome 7q11.23-q21.11. *Am J Hum Genet* **83**, 106-111 (2008).

47. E. Bruneau, M. Akaaboune, The dynamics of the rapsyn scaffolding protein at individual acetylcholine receptor clusters. *J Biol Chem* **282**, 9932-9940 (2007).
