# Supplementary material for: Systematic Prediction of Scaffold Proteins Reveals New Design Principles in Scaffold-Mediated Signal Transduction
Source: PLoS Comput Biol. 2015 Sep 22;11(9):e1004508. doi: 10.1371/journal.pcbi.1004508 (PMC4578958; doi:10.1371/journal.pcbi.1004508)
Supplement: S3 Table — (DOCX) [file pcbi.1004508.s010.docx]

S3 Table.Validation hit list of predicted scaffold proteins.

| **Predicted Scaffold** | **Kinase** |
| --- | --- |
| PIN1 | CSNK2A1 |
| ATF2 | CSNK2A1 |
|  | MAPK9 |
