# Supplementary material for: Systematic Prediction of Scaffold Proteins Reveals New Design Principles in Scaffold-Mediated Signal Transduction
Source: PLoS Comput Biol. 2015 Sep 22;11(9):e1004508. doi: 10.1371/journal.pcbi.1004508 (PMC4578958; doi:10.1371/journal.pcbi.1004508)

S4 Table. List of *PIN1*and *ATF2* meditated phosphorylation events. Each array was manually aligned and scored. Final hits that were identified were replicated, not present in control arrays, and had F/B ratios >1.5. Proteins in bold were hit with both *ATF2* and *PIN1*.


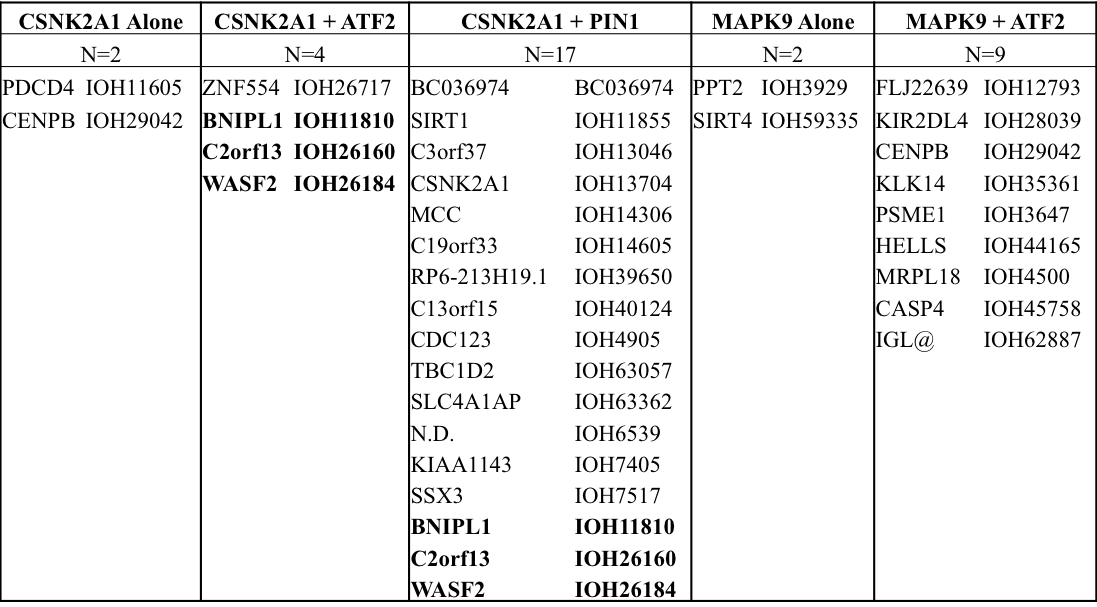

Supplement: S4 Table — Each array was manually aligned and scored. Final hits that were identified were replicated, not present in control arrays, and had F/B ratios >1.5. Proteins in bold were hit with both ATF2 and PIN1. (DOCX) [file pcbi.1004508.s011.docx]
